# Children’s exposure to physical abuse from a child perspective: A population-based study in rural Bangladesh

**DOI:** 10.1371/journal.pone.0212428

**Published:** 2019-02-19

**Authors:** M. Atiqul Haque, Staffan Janson, Syed Moniruzzaman, A. K. M. Fazlur Rahman, Syed Shariful Islam, Saidur Rahman Mashreky, Ulla-Britt Eriksson

**Affiliations:** 1 Public Health Sciences, Department of Health Sciences, Karlstad University, Karlstad, Sweden; 2 Department of Women’s and Children’s Health, Uppsala University, Uppsala, Sweden; 3 Risk and Environmental Studies, Department of Environmental and Life Sciences, Karlstad University, Karlstad, Sweden; 4 Center for Injury Prevention and Research, Bangladesh, New DOHS, Mohakhali, Dhaka, Bangladesh; 5 Bangladesh University of Health Sciences, Darus Salam, Mirpur, Dhaka, Bangladesh; 6 Department of Public Health and Informatics, Bangabandhu Sheikh Mujib Medical University, Shahbagh, Dhaka, Bangladesh; University of Texas Medical Branch at Galveston, UNITED STATES

## Abstract

**Background:**

Although child physical abuse (CPA) is considered as a major global public health problem, it has not yet been recognized as such in Bangladesh. Very few studies have assessed the prevalence and victims’ characteristics of multiple forms of CPA.

**Objective:**

This population-based study assessed the prevalence of CPA committed by adults in a rural area of Bangladesh and examined its association with demographic and socio-contextual factors.

**Methods:**

Data were obtained using ISPCAN Child Abuse Screening Tool for Children (ICAST-C) in a random sample of 1416 children (49% girls, 51% boys) aged 11 to 17 years by face-to-face interviews during March-April 2017. The response rate was 91.5%. To estimate predictors of CPA, physical abuse was categorized into frequent and less frequent groups.

**Results:**

The prevalence of at least one form (≥ 1), two forms (≥2) and three or more forms (≥ 3) of CPA were estimated approximately to 99%, 95% and 83% in their lifetime and 93%, 79%, and 57% in the past year respectively. Hitting (except on buttocks), standing/kneeling and slapping were the most common physical abuse whereas given drugs or alcohol, pinched, burned or scalded, beaten-up and locked up were less reported. Female children were faced severe forms of CPA more than that of males. Male children, younger age groups, witnessing adults using weapons at home, bullied by siblings and low level of maternal education were found to be significant risk factors for both ≥ 1 form and ≥ 2 forms of frequent CPA whereas adding also adult shouting in a frightening way was found as a significant risk factor for ≥ 2 forms of frequent CPA.

**Conclusion:**

Self-reported prevalence of CPA is extremely common in the Bangladeshi rural society. The prevalence was associated with demographic and socio-contextual characteristics of the children such as being younger, witnessing domestic violence and maternal low education. The findings provide evidence to support parents and policy-makers to take effective measures to implement policy and programme on alternative up-bringing methods and creating awareness of negative effects of CM which in turn help Bangladesh to line up with UN Convention on the Rights of the Child, which the country signed in 1990.

## Introduction

Child maltreatment (CM) is a global public health problem with immediate and long-term health consequences [1,2]. Although CM has been existing since time immemorial, recently, this issue has got a widespread attention by policy-makers in the developed countries and several steps have been taken to prevent it [3]. In 1989, United Nations adopted the Convention on the Rights of the Child (CRC) to protect the rights of children worldwide and this has been ratified by 195 countries, which makes it the most widely ratified human rights treaty in the world [4]. Of special interest is Article-19 of CRC, which states that every country is obliged to protect children from all kinds of CM [5]. Sweden was the first country to introduce a corporal punishment ban and has in October 2018 been followed by 54 countries, whereas only four countries in Asia, namely Israel, Turkmenistan, Mongolia and Nepal have banned corporal punishment [6].

The prevalence of CM varies widely by countries. However, it is difficult to make comparisons among countries due to definitional inconsistencies, methodological variations in conducting research, varied types of maltreatment, children’s status and reporting methods [2,7]. Despite these discrepancies, it is evident that CM is a serious social and public health problem, especially in low-income countries [8,9]. It is also a problem that child-rearing practices viewed as acceptable in one culture are assessed as abusive in another culture. In the Indian subcontinent parental belief regarding particular forms of harsh treatment is considered necessary in the best interest of children [10,11]. Besides, a dearth of valid data regarding CM in different countries makes it difficult to design and implement policies, plans and programs to address this issue effectively [12,13].

The World Health Organization reveals that while 25–50% of all children are physically abused, 20% girls and 5–10% boys are abused sexually worldwide [14]. An Indian study reported that 75.5% and 78.5% school-going children in Kerala were physically abused during the past year and lifetime respectively [15]. Similarly, high prevalence rates of CM were observed in a suburban community in Sri Lanka (76%) [16] and in Punjab, Pakistan (80%) [17]. Further, in Vietnam, 73% of the primary caregivers practiced violent discipline towards their children [18]. Many children witness violence regularly. A UN report estimates that 41–88 million children witness violence at home in South Asia, making the region to have the world’s highest figures in this category [2].

There are few studies on CM published in scientific international journals from Bangladesh [19,20], but there are numerous case reports on different types of violations towards children [21]. Most of the information regarding CM in Bangladesh comes to the public by news media or through government or non-government organizational reports. Though some media reports on CM played a significant role in issuing the high court directive banning of corporal punishment at school in 2010, media coverage of child issue is still negligible and it constitutes only 3% of the total media reports [22]. Despite low reporting rate, we found that print media tends to report severe physical and sexual abuse, where fatal cases get more prominence [23]. According to a recently published report, 82.4% of children in Bangladesh aged 1–14 years experienced either physical or psychological abuse [24].

CM stems from a series of interrelated factors and identifying the definitive causes is complicated. Belsky modified the Bronfenbrenner’s ecological model of child development to conceptualize the risk factors of CM and describes that cultural, communal, familial and personal factors intersect to increase the likelihood of CM [25]. Earlier studies focused on poverty, overcrowding households, low parental education, young parents, parental unrealistic expectations about child development, parental substance abuse, social instability, younger children and childhood maltreatment history of parents as determinants of CM [26,27]. Hadi [19] reported that poverty, being a male child and low parental education were related to higher CM in Bangladesh. The powerlessness and the subordinate position of children in the Bangladeshi society were explanations found to children’s experiences and perceptions on CM in a recent study by our team [11].

In order to address the CM issue properly and to take an effective approach in preventing CM, it is essential to understand the magnitude of the problem and demographic and socio-cultural characteristics of the victims. A dearth of reliable data on the prevalence and risk factors of child physical abuse (CPA) in Bangladesh is one of the main hindrances to address this issue properly. Therefore, the current study was designed to investigate the prevalence and risk factors for physical abuse of children from a child perspective.

## Methodology

### Study setting

This cross-sectional study was conducted in Raiganj upazila (sub-district) of Sirajganj district, an agro-based rural area, situated in the north-western part of Bangladesh and approximately 150 km away from the capital city, Dhaka. The Centre for Injury Prevention and Research, Bangladesh (CIPRB) has been maintaining an injury and demographic surveillance system in three unions (administrative unit under upazila) of Raiganj since 2006 and since then socio-demographic and injury-related data of all residents of this area are updated twice a year. In 2017, the total population and number of households in this area were 146,828 and 35,071 respectively. Each of the persons of this surveillance area has a unique identification number and this number was the base of our sampling frame.

### Participants

In total 1,547 children aged 11–17 years were selected as study sample using simple random sampling procedure. The sample size was estimated based on a prevalence of CM 82.4% from a previous study [24], an absolute precision of 2% and a confidence level of 95% according to the methodology described by Lwanga & Lemeshow [28] considering a response rate of 90%. A complete list of household addresses of all sampled children was prepared beforehand. Children over 11 years were chosen since they were considered to understand concepts and have the ability to elaborate on their own life situation. It is an age where they normally understand informed consent [29,30]. The survey took place in March and April 2017. The research team interviewed 1,416 children from separate households with a response rate of 91.5%.

### Tool

International Society for the Prevention of Child Abuse and Neglect (ISPCAN) Child Abuse Screening Tool for Children (ICAST-C) was used in this study to evaluate the prevalence of physical abuse of children [29]. Since 2004, ISPCAN has been working together with UNICEF in developing three questionnaires, one for parents (ICAST-P), another for young adults (ICAST-R) and the third for children (ICAST-C). In 2014, ICAST-C was updated and prepared as a single tool combining previously used Institutional and Home versions [29]. This tool has been successfully serving as a common instrument to measure the prevalence of different forms of CM worldwide and it is possible to make a comparison of CM data across cultures, time or between studies.

There are a number of well-performed studies globally using ICAST-C [15,31,32]. In the Asian context, this tool was found reliable and valid in different cultural settings [33].

In the Bangladeshi socio-cultural context, 17 questions of ICAST-C were found related to CPA and these questions were asked to the respondents with reference to the past year. All questions had multiple choice responses: “once a week or more often”, “several times a month”, “about once a month”, “several times a year”, “once or twice a year”, “not in the past year, but it has happened before”, “never in my life” and “no answer”. The children were supposed to select only one of these options. If children would respond positively, they were asked to identify the offender as “adult male”, “adult female”, “child/adolescent male”, and “child/adolescent female”. In this study, we considered adult offender only. Besides these, participants were also asked about safety feeling, bullying and family violence.

Further, participant’s living situation, birth order, educational status, marital status, occupation, parental education level and the following socio-economic data: household assets such as table, chair, watch, computer, electricity supply, refrigerator, television, radio, mobile phone, bicycle, air conditioners, number of farm animals, agricultural land etc.; dwelling characteristics; source of drinking water; toilet facilities and cooking fuel were collected. The number of the questions followed the original ICAST-C questionnaire and all additional questions were numbered in a distinct way.

The original English ICAST-C tool was translated into Bengali (the official and common people’s language of Bangladesh) and back-translated for comparison. Standard translation procedures were followed by bilingual experts and the Bengali version was tested by two boys and two girls aged 11-17- years and was found understandable.

Focus Group Discussions (FGDs) with professionals, rural parents, male and female children aged 11–17 years were organized to sensitize the vocabulary and thinking patterns of the population and to accustom with the content area of the study as suggested in ICAST manual. The FGDs were audio-taped, transcribed verbatim and necessary modifications were made in the data collection tool.

### Procedure of data collection

Training: We developed an interviewer manual in the local language to be used for implementation of the study with attention to consent, ethics, and data collection technique in electronic devise. A three-day training session was organized where the five female data collectors were trained to introduce themselves, explain the purpose of the study, obtain informed consent, administer the data collection tool, ask about abuse in a non-judgmental way, preserve confidentiality, and recognize possible negative reactions and respond properly. As part of their training, they interviewed 17 children aged 11–17 years outside the study area to be familiar with the interview process and also to check for any inconsistency in the tool.

Data collection: The whole study area was divided into 19 blocks based on data collector’s assignment. In order to collect data, the data collectors visited the households of the children. The interviews were made in a quiet place with a face to face approach to collect the data.

The questionnaire was designed in REDCap (Research Electronic Data Capture) for data collection. REDCap is a web-based, secure, reliable free software application designed for collecting and managing research data developed at Vanderbilt University [34]. The collected data were exported to the Statistical Package for Social Sciences (SPSS) version 24 for analysis.

### Data analysis

Descriptive analysis was done on participants’ socio-demographic variables, family characteristics and family violence. Frequencies and percentages were calculated as summary measures for the qualitative variables, and arithmetic mean and the standard deviation was used to describe the quantitative variables. Principal component analysis was adopted to construct wealth index based on household socio-economic data and dwelling characteristics [35]. The frequency of various forms of CPA was reported. The past year prevalence of CPA was calculated by dichotomizing all forms of abuse based on any vs. no exposure in the past one year. For lifetime prevalence, forms were dichotomized based on any vs. no exposure in the past year or ever. Prevalence was shown in percentages with 95% confidence interval (CI).

We further categorized all 17 forms of CPA into frequent (≥3 occurrences/year) and no frequent (none to ≤2 occurrences /year) groups. The frequent category was based on the following responses: “once a week or more often”, or “several times a month”, or “about once a month”, or “several times a year”, and no frequent category was based on the followings “once or twice a year”, “not in the past year, but it has happened before” or “never in my life”. Univariate and multivariate logistic regression analyses were used to determine which of the independent variables predicted the occurrence of “frequent” CPA.

Independent variables that demonstrated a significant (p < .05) risk factor (expressed in crude OR) in the univariate model were selected for further analysis in a binary logistic regression model in order to identify independent risk factors (expressed in adjusted OR) for the occurrence of frequent CPA. In this regard, all significant variables among gender, age, education, living arrangement, safety feeling at home, parental education, socio-economic status (SES) base on wealth index, bullied by siblings and witnessing family violence among adults like shouting, physical violence and using weapon were entered simultaneously in the adjusted analyses. A P-value less than 5% was considered as a level of significance.

### Ethical considerations

The questionnaire has some sensitive questions and asking them might be emotionally disturbing. Therefore, the ethical issues were carefully considered in the consent form, to ensure voluntary participation and to maintain confidentiality. Confidentiality was maintained keeping the interviewee anonymous with no identifying information on the questionnaire. Furthermore, after every interview, the child was asked how (s)he felt and was encouraged to talk to a trusted person if the interview evoked unpleasant feelings. However, such a situation did not occur during the data collection.

Children and their parents were informed that the study was about violence against children and the participation was non-paid and voluntary. Children were invited for an interview with a data collector. Prior to the interview, parental written consent for children’s participation and assent from children were obtained as per the requirement of local ethical boards.

The research protocol was approved by the ethical review board of CIPRB (memo number: CIPRB/ERC/2016/14, Date: 29 June 2016) and Bangabandhu Sheikh Mujib Medical University (BSMMU), Bangladesh (memo number: BSMMU/2017/3228 (A), Date: 1^st^ April 2017). Ethical permission was also obtained from the Regional Ethical Review Board in Uppsala, Uppsala University, Sweden (Dnr 2016/520, Regionala etikprövningsnämnden i Uppsala).

## Results

### Characteristics of the children

The survey was completed by interviewing 1,416 children aged 11 to 17 years. Their median age was 14.4 years (14.5 for boys and 14.2 for girls), and their demographic and socio-cultural characteristics are summarized in Table 1. The most common family size was with two children (37%) followed by three children (34%) and most interviewees (36%) were the eldest child of the family. In total 86% of the interviewed children were students at the time of the interview and more than 10% were working mostly with household work (6.8%) and agricultural work (1.7%). Almost all children resided with their parents or family members (94.3%). Among all children, almost 12% were married (10% girls, 1.7% boys). The largest proportion of fathers had no formal education (37.1%) followed by primary level education (33.0%) whereas mothers were mostly educated up to the primary level (47.5%). A few parents had a completed graduation degree (2.2% of fathers and 0.7% of mothers).

**Table 1:** Participants’ demographics and socio-contextual details, n (1416).

Characteristics	No. of participants	% of sample
**Gender**		
Male	726	51.3
Female	690	48.7
**Age (year)**		
11–14	838	59.2
15 and above	578	40.8
**Birth order of children**		
1^st^ baby	511	36.1
2^nd^ baby	421	29.7
3^rd^ baby	305	21.5
≥4^th^ baby	179	12.6
**No. of children in family**		
One	72	5.1
Two	523	36.9
Three	483	34.1
≥Four	338	23.9
**Education level of children**		
No schooling	9	.6
Primary (1–5 grade)	462	32.6
Secondary (6–10 grade)	928	65.5
Higher secondary (11–12 grade)	17	1.2
**Occupation of the children**		
Student	1224	86.4
Service	12	.8
Household work	96	6.8
Agricultural work	24	1.7
Idle/not working	16	1.1
Student but part time service	8	.6
Others	36	2.5
**Marital status**		
Married	81	5.7
Unmarried	1333	94.1
Divorced	2	.1
**Living arrangement**		
Lives with both parents	1293	91.3
Lives with biological mother only	25	1.8
Lives with relatives	78	5.5
Lives with biological and step parents	17	1.2
Lives in boarding house	3	.4
**Father’s education level**		
No schooling	528	37.3
Primary School (1–5 Grade)	469	33.1
Secondary School (6–10 Grade)	184	13.0
Higher Secondary School (11–12 Grade)	76	5.4
Graduate and above	32	2.2
Others	127	10
**Mother’s education level**		
No schooling	374	26.4
Primary School (1–5 Grade)	675	47.7
Secondary School (6–10 Grade)	264	18.6
Higher Secondary School (11–12 Grade)	28	2.0
Graduate and above	10	.7
Others	65	4.6
**Adult at home shouted in a frightening way**	784	55.4
**Witnessed adult’s violence (home)**	852	60.2
**Witnessed adults at home use weapons**	142	10.0
**Bullied by siblings**	344	24.3
**Always safe feeling at home**	1353	95.6

### Prevalence of CPA

Table 2 shows a summary of the prevalence rates of CPA both in the past year and over the lifetime. The past year prevalence rates of at least one form (≥1), at least two forms (≥2) and three or more forms (≥3) of physical abuse were 92.8%, 79.1%, and 57.1% respectively where these rates were 98.7%, 94.9% and 83.4% respectively over the lifetime.

**Table 2:** Prevalence of the past year, lifetime and frequent child physical abuse (n = 1416).

	Past year		Lifetime		Frequent physical abuse				
Physical Abuse							Gender n (%)		P value
	Frequency	Percentage (95% CI)	Frequency	Percentage (95% CI)	Frequency	Percentage (95% CI)	Male	Female	
At least one form (≥1)	1314	92.8 (91.5–94.2)	1398	98.7 (98.1–99.3)	1106	78.1 (75.9–80.2)	583 (80.3)	523 (75.8)	0.04
At least two forms (≥2)	1120	79.1 (77.0–81.2)	1344	94.9 (93.8–96.1)	657	46.4 (43.8–49.0)	368 (50.7)	289 (41.9)	0.001
Three or more forms (≥3)	808	57.1 (54.5–59.7)	1181	83.4 (81.5–85.3)	362	25.6 (23.3–27.9)	213 (29.3)	149 (21.6)	0.001

Children experienced a mean of 2.84 (SD 1.6) victimizations during the past year and 4.28 (SD 2.01) victimizations over their lifetime. Male children were subjected to more physical victimizations than females for both past year and over the lifetime (p values<0.001).

### Prevalence of frequent CPA

After analysing all forms of abuse of frequent and no frequent categories, we have found approximately 78.1%, 46.4% and 25.6% of children experienced at least one form, at least two forms and at least three forms of frequent physical abuses respectively. Male children were abused significantly more than that of female children (Table 2).

### Characteristics of physical abuse against children

Table 3 summarizes the lifetime and the past year prevalence rates of different forms of CPA. Hit elsewhere except on buttocks, standing/kneeling and slapping on the head or face were reported as common physical abuse. Female children experienced severe forms of physical abuse and results show significantly higher rates of the past year for hair pulled and lifetime shaken, choked, burned or scalded than that of males. During lifetime, though non-significant, female children are also more subjected to other severe forms of physical abuse like kicked, beaten-up, slapped in head or face. No child had experienced physical abuse by means of putting hot pepper, soap or spicy food in their mouth.

**Table 3:** Percentage distribution of children’s reported experience of physical abuse by gender.

	Lifetime			Past year			Frequent physical abuse		
Forms of abuse	Total	Female	Male	Total	Female	Male	Total	Female	Male
Kicked (d18)	23.2	23.6	22.9	8.2	7.2	9.1	2.5	2.3	2.8
Shaken (d19)	15.7	18.0	13.6*	4.9	4.8	5.1	1.5	1.4	1.5
Slapped in head or face (d20)	78.2	78.3	78.2	56.1	55.1	57.0	38.3	37.0	39.7
Hit on the head with knuckles (d21)	12.1	8.1	15.8*	6.4	4.2	8.4*	2.8	2.2	3.4
Spanked (d22)	16.5	16.8	16.1	7.9	7.8	8.0	4.1	3.5	4.7
Hit on the buttocks with object (d23)	18.4	13.3	23.3*	10.3	6.1	14.3*	4.1	1.7*	6.3
Hit elsewhere except on the buttocks with object (d24)	89.8	87.7	91.9*	78.0	75.8	80.2*	58.8	56.4	61.0
“Beaten-up” (d25)	7.8	8.0	7.6	2.5	2.2	2.8	1.3	1.2	1.5
Choked (d26)	11.7	13.6	9.8*	0.6	0.9	0.4	0.1	0.1	0.1
Burned or scalded (d27)	4.6	7.2	2.1*	1.6	2.2	1.1	1.4	1.9	1.0
Locked up (d29)	8.7	4.9	12.3*	3.2	1.6	4.7*	2.1	0.7*	3.4
Ear twisted (d30)	39.8	25.9	53.0*	26.1	16.5	35.3*	11.6	6.5*	16.4
Hair pulled (d31)	22.5	26.1	19.1*	14.3	16.2	12.5*	5.2	5.2	5.2
Pinched in (d32)	4.5	5.5	3.6	3.5	4.5	2.6	0.6	0.6	0.7
Stand /kneel for punishment (d33)	74.1	71.9	76.2	60.6	58.0	63.1*	29.5	25.2*	33.6
Given drugs or alcohol (d36)	0.4	-	0.8*	.1	-	0.3	0.1	-	0.1

*p value < .05

### Risk factors

Table 4 shows the predicting factors associated with the occurrence of frequent CPAs. 4^th^ and 5^th^ columns show the crude ORs and adjusted ORs respectively for the occurrence of ≥1 form of frequent CPA where 7^th^ and 8^th^ columns report the cORs and aORs for the occurrence of ≥2 forms of frequent CPA respectively. Our result shows that children of male gender, aged 14 years and below, and the children who witnessed adult’s using of weapon to hit other at home, was bullied by siblings and had a mother of education level lower than secondary were significantly associated with the occurrence of ≥1 form of frequent CPA after adjusting for other variables.

**Table 4:** Predicting factors for frequent CPA experience.

	No frequent CPA, n (% of children in respective group)	Frequent CPA (≥1 forms), n (% of children in respective group)	cOR^†^	aOR^‡^ (95% CI)	Frequent CPA (≥2 forms), n (% of children in respective group)	cOR^†^	aOR^‡^ (95% CI)
**Gender**							
Male	143 (46.1)	583 (52.7)	1.30 (1.01–1.68)*	1.41 (1.08–1.83)*	368 (56.0)	1.49 (1.13–1.95)**	1.75 (1.29–2.36)***
Female	167 (53.9)	523 (47.3)	Reference	Reference	289 (44.0)	Reference	Reference
**Age (year)**							
11–14	156 (50.3)	682 (61.7)	1.59 (1.23–2.05)***	1.71 (1.29–2.26)***	420 (63.9)	1.75 (1.33–2.30)***	1.98 (1.44–2.71)***
≥15	154 (49.7)	424 (38.3)	Reference	Reference	237 (36.1)	Reference	Reference
**Children’s education level**							
Up to primary	83 (26.8)	388 (35.1)	1.48 (1.12–1.96)**	1.06 (.78–1.44)	249 (37.9)	1.67 (1.24–2.25)**	1.14 (0.81–1.60)
Secondary	227 (73.2)	718 (64.9)	Reference	Reference	408 (62.1)	Reference	Reference
**Living arrangement**							
Biological parents	296 (95.5)	1022 (92.4)	.58 (.32–1.03)		603 (91.8)	0.53 (0.29-.97)*	.50 (.26-.99)*
Others arrangement	14 (4.5)	84 (7.6)	Reference		54 (8.2)	Reference	Reference
**Safe feeling at home**							
Not always	7 (2.3)	56 (5.1)	2.31 (1.04–5.12)*	2.05 (.91–4.64)	29 (4.4)	1.9 (.87–4.61)	
Always	303 (97.7)	1050 (94.9)	Reference	Reference	628 (95.6)	Reference	
**Adult at home shouted in a frightening way**							
Yes	148 (47.7)	636 (57.5)	1.48 (1.15–1.91)**	1.25 (.96–1.63)	434 (66.1)	2.13 (1.62–2.81)***	1.68 (1.25–2.26)**
No	162 (52.3)	470 (42.5)	Reference	Reference	223 (33.9)	Reference	Reference
**Witnessed adults physical violence (at home)**							
Yes	158 (51.1)	693 (62.7)	1.59 (1.24–2.05)***	1.27 (.97–1.67)	415 (63.2)	1.63 (1.24–2.14)***	1.25 (.92–1.71)
No	151 (48.9)	413 (37.3)	Reference	Reference	242 (36.8)	Reference	Reference
**Witnessed adults at home use weapons**							
Yes	7 (2.3)	135 (12.2)	6.02 (2.79–13.01)***	4.92 (2.25–10.77)***	96 (14.6)	7.41 (3.40–16.16)***	5.60 (2.50–12.55)***
No	302 (97.7)	971 (87.8)	Reference	Reference	561 (85.4)	Reference	Reference
**Bullied by siblings**							
Yes	34 (11.0)	310 (28.0)	3.16 (2.16–4.62)***	2.95 (1.99–4.36)***	211 (32.1)	3.84 (2.60–5.68)***	3.59 (2.37–5.43)***
No	275 (89.0)	796 (72.0)	Reference	Reference	446 (67.9)	Reference	Reference
**Father’s education level**							
Below secondary level	229 (73.9)	888 (80.7)	1.47 (1.1–1.98)*	1.01 (.69–1.49)	520 (79.1)	1.34 (.98–1.84)	
Secondary and above	81 (26.1)	214 (19.3)	Reference	Reference	137 (20.9)	Reference	
**Mother’s education level**							
Below secondary level	220 (71.0)	893 (80.7)	1.72 (1.29–2.29)***	1.48 (1.01–2.15)*	530 (80.7)	1.71 (1.25–2.33)**	1.62 (1.11–2.36)*
Secondary and above	90 (29.0)	213 (19.3)	Reference	Reference	127 (19.3)	Reference	Reference
**SES composite wealth index**							
Lower	107 (34.5)	459 (41.5)	1.39 (.99–1.96)		291 (44.3)	1.51 (1.05–2.19)*	.79 (.49–1.26)
Middle	134 (43.2)	434 (39.2)	1.05 (.75–1.46)		242 (36.8)	1.01 (.70–1.44)	.95 (.49–1.15)
Upper	69 (22.3)	213 (19.3)	Reference		124 (18.9)	Reference	Reference

*P < .05

**P < .01

**P < .001

cOR = unadjusted odds ratio; aOR = adjusted odds ratio.

†Odds ratio with “No frequent CPA” as the comparison group.

‡All significant variables in univariate analysis in the table were entered simultaneously in multivariate logistic regression model in order to calculate aOR.

Similarly, male children, lower age group, bullying by siblings, maternal low education level and child’s witnessing domestic violence like adult’s shouting in a frightening way, and use of weapons were found to be significant predictors for the occurrence of ≥2 forms of frequent CPAs. The living arrangement with biological parents was found to be significant protective factors (OR 0.50; 95% CI 0.26–0.99) against the occurrence of ≥2 forms of frequent CPAs.

## Discussion

This study of Bangladeshi rural children analysed the prevalence of physical maltreatment by adults and examined associated demographic and socio-cultural risk factors. The findings of this study indicate that physical abuse by adults among rural children is extremely common, with an estimation of 93% and 99% of all children experiencing at least one form of physical abuse within the past 12 months and in their lifetime respectively.

Studies using the ICAST tool have found high prevalence rates of physical abuse. However, our findings are much higher compared to the findings of most of these studies. Using ICAST tool, Ribeiro et al [32] reported 85.4% prevalence of physical abuse among school-going children in Brazil. Similarly, Kumar et al [15] found that 75.5% school-going children were physically abused during the past year and 78.5% during lifetime respectively in Kerala, India. Likewise, 58% of Saudi Arabian children were found experiencing physical abuse in the past year [36] and 58% Turkish children during their lifetime [37]. Using the same tool in a multi-country study in the Balkan region also reported high prevalence rates of CPA [38].

Survey data reported varied rates of physically abused children worldwide [39]. Meta-analyses revealed that the prevalence of physical abuse among Chinese children ranging from 26.2% [40] to 36.6% [41] whereas the worldwide prevalence was 22.6% [39]. This variation is partly due to differences in methodology, definition of CM and the ages of the study population.

Stoltenborgh et al [39] reported that studies using a comprehensive definition of CPA, estimating physical abuse across childhood (0–18 years) and using several questions on physical abuse tended to give the highest prevalence rates. They also acknowledged that a high tolerance of corporal punishment in Asian culture might lead to a higher prevalence. In line with these explanations, our reported high prevalence of physical abuse might be due to the comprehensiveness of the ICAST tool and cultural acceptance of physical punishment to discipline children. A study in the Southeast Asian context revealed that for managing children’s behaviour and helping them to become responsible adults, punishment is considered as an effective measure [42]. In our previous study, we found that CM is also associated with hoping for better academic achievement [11].

In accordance with Belsky’s ecological model it is expected that societal and cultural factors at macro-system might have significant association with the high rate of child physical abuse in Bangladesh. The country got its independence from Pakistan in 1971 after a violent war in which it was claimed that some three million people were killed and 0.2 million women were raped [43]. Since its independence, Bangladesh witnessed violence between political parties and government forces in their dealings with one another where extra-judicial killings are frequently reported in media. Several incidents of violent attacks in 2016 against secular writers and bloggers, academics, foreigners, gay rights activists and religious minorities have added a new dimension to this violence. There are also several violent cases with fatal consequences reported in the national dailies almost every day [23].

Besides, thousands of people die every year due to natural calamities and millions are affected by arsenic contamination. Within this violent and disaster-prone society, high rate of poverty, intimate partner violence (IPV) and violence against women increase stress on the whole family, which act as vulnerable factors that serve to potentiate child maltreatment in a larger scale [44].

Bangladeshi people are thus accustomed to witnessing violence and its tolerance can be a contributing factor to the high prevalence of child maltreatment of the country.

Since its independence Bangladesh Government has endorsed some laws and acts to promote child rights. However, there is very little implementation and awareness among the population. After the ratification in 1990 of UNCRC it took more than two decades to get a child act in 2013. The government is yet to promulgate laws banning corporal punishment in all settings and laws against child maltreatment to ensure child rights and wellbeing.

Of the 17 physical abusive events of the present survey, “hit elsewhere except on buttocks”, “standing/kneeling” and “slapping on the head or face” were reported as common physical abuses whereas “giving drugs or alcohol” was the least. Similarly, Indian adolescent school-going girls mainly experienced slapping or hitting [45]. Slapping was also found as one of the most common forms of physical punishment experienced by the children of Pakistani immigrants in the UK [46]. Putting hot pepper in the mouth was one of the most common types of physical abuse experienced by Turkish children [37] whereas no one in our study reported this form of abuse.

Our results suggested that boys experienced significantly more physical abuse than girls, similar to—studies in China [47], Cyprus [48] and India [49]. In a Pakistani study Lakhdir et al. [50] stated that a male child is fivefold more prone to be maltreated compared with the female child. In educational institutions of Bangladesh, girls received least physical punishment (81%) while boys received the most (98%) [51]. However, our findings somewhat different from what Stoltenborgh et al [39] and Flynn-O’Brien et al [52] have found where gender differences were absent. According to 2006 survey of ILO, some 420,000 children in Bangladesh are domestic workers, mostly girls aged 6 to 17 years old, though this number is assumed much higher now. The majority of domestic working children are from impoverished rural families, who have migrated to urban areas where indiscriminate violation of their rights are frequently reported in media. These young girls sometimes experience severe forms of inhumane physical torture like being hit in the head against the wall, burnt by lit cigarettes or hot metal objects, severely beaten by sticks or metal rods [53]. In consistence with this, female children of our study were found to be more subject to severe forms of physical abuse.

One of the reasons behind more physical abuse against male children in the Bangladeshi society could be which Benbenishty et al [54] reported, that males are more likely than females to have discipline problems and they respond better to physical than verbal intimidation.

Age of children is an important risk factor. Our study revealed that younger age group children were significantly more abused physically than elderly children. This finding is in line with other regional studies conducted in Pakistan [50] and Vietnam [55]. Hunter et al [56] stated that disciplining young children in the hope of achieving a better future shows the tendency of Indian mothers to legitimize physical abuse. Beside this, older children gain some physical developments such as increasing body weight, increasing muscle and other adult-like appearances which protect them from being physically punished by adults. This explanation is congruent with the finding of Lakhdir et al [50]. It is also expected in the Bangladeshi society that older children at work are less abused since they are considered as economically valuable in poor families.

Home environment and family relationship are important factors for child development. Our findings of associations between CPA and witnesses of family violence are in line with studies from other countries revealing a strong relationship between witnessing domestic violence and CPA and other forms of CM [57–59]. In the South Asian context, Hunter et al [56] showed the occurrence of domestic violence at home to double the risk of child abuse in an Indian sample whereas in a Pakistani sample this risk increased nine-fold [50]. The possible explanation of this co-occurrence happens due to family crises and stressful circumstances where economic and parenting stress leads to violence across family dyads (parent-child, parent-parent) [60,61]. People who live in rural areas of Bangladesh have a high level of stressful economic situation where approximately 35% live under the poverty line ($2USD) [62].

Our result substantiates that low or non-formal maternal education is an important risk factor for CPA in the Bangladeshi rural context. This finding is consistent with study results conducted in India [56] and Iran [63] that showed low maternal education to be related to higher CM.

We estimated different significant risk factors for CPA among which two levels of Belsky’s ecological model, mainly individual (gender, age, and parental educational level) and family (different types of family violence) have been addressed [25]. Future studies need to further address community and societal level risk factors of this model.

### Study limitations and strengths

The data on the prevalence of CPA were collected in a cross-sectional study and can therefore only indicate potential associations between CPA and other demographic and socio-contextual variables and cannot provide any evidence of cause and effect. Secondly, the study was based on the participants’ self-reported victimization, with a lack of independent verification, which may cause some self-reporting bias. Thirdly, the data were collected in an isolated place for ethical reasons, but absolute confidentiality was not always possible to maintain due to overcrowding surroundings and parental interference in interview. Fourthly, due to the high illiteracy data were collected by face to face interviews and asking children about sensible matters such as maltreatment experiences could influence their response due to feelings of being exposed. Fifthly, our data collection tool was ICAST-C, which had no scope to measure other types of violence than committed by adults.

One strength with the study was that samples were obtained from a surveillance area, where every individual has a unique identification number and based on these numbers a simple random sampling procedure was employed. The large sample size, population based sample and high response rate are other strengths. Further, software-based data collection system keeps the missing data in a negligible amount. The use of an internationally validated tool such as the ICAST-C questionnaire gives possibilities to make comparisons with other studies and was a worthy tool to make assessment of CM in a low-income country context.

### Policy implication

Our study findings put forward some key questions for further research. The results show that almost every child experienced physical abuse. A policy should be adapted to influence adults’ awareness of negative effects of CPM to get a deeper understanding of child development and changing of attitudes and behaviour. Several risk factors of CPA revealed in this study such as being male, younger, bullied by siblings and witnessing family violence might help policy-makers to create increased protective laws and devices.

## Conclusion

This is the first population-based study in a rural area of Bangladesh using the ICAST-C tool. Prevalence of children’s self-reported physical abuse in all forms was extremely high in the Bangladeshi rural society. The prevalence of CPA was associated with demographic and socio-cultural characteristics of the children, addressing individual and family-level risk factors such as being younger, witnessing domestic violence and maternal low education.

## References

[1] Middlebrooks JS, Audage NC. The effects of childhood stress on health across the lifespan. Atlanta; GA:Centers for Disease Control and Prevention, National Center for Injury Prevention and Control; 2008. Available from: http://health-equity.lib.umd.edu/932/1/Childhood_Stress.pdf Cited 13 April 2018

[2] Pinheiro PS. World report on violence against children. Geneva, United Nations; 2006. 387 p. Available from: https://www.unicef.org/violencestudy/I. World Report on Violence against Children.pdf Cited 13 January 2017

[3] Crosson-Tower C. Understanding child abuse and neglect 9th ed Dodge A, editor. Upper Saddle River: Pearson; 2014.

[4] Schapper A. Children’s rights implementation as a multi-level governance process. Hum Rights Q. 2017;39(1):104–29. 10.1353/hrq.2017.0003

[5] UN General Assembly. Convention on the Rights of the Child. UN General Assembly resolution 44/25. 1989. Available from: http://www.ohchr.org/Documents/ProfessionalInterest/crc.pdf Cited 24 Feb 2018.

[6] Global Initiative to End All Corporal Punishment of Children. Countdown to universal prohibition 2018. Available from: http://www.endcorporalpunishment.org/ Cited 11 Oct 2018

[7] Fergusson DM, Horwood LJ, Boden JM. Structural equation modeling of repeated retrospective reports of childhood maltreatment. Int J Methods Psychiatr Res. 2011;20(2):93–104. 10.1002/mpr.337 21495111 PMC6878375

[8] Akmatov MK. Child abuse in 28 developing and transitional countries-results from the multiple indicator cluster surveys. Int J Epidemiol. 2011;40(1):219–27. 10.1093/ije/dyq168 20943933

[9] Jud A, Fegert JM, Finkelhor D. On the incidence and prevalence of child maltreatment: a research agenda. Child Adolesc Psychiatry Ment Health 2016;10:17 10.1186/s13034-016-0105-8 27303442 PMC4907083

[10] Korbin JE, editor. Child abuse and neglect: Cross-cultural perspectives. Oakland, CA: Univ of California Press; 1983.

[11] Haque MA, Janson S, Moniruzzaman S, Rahman AKMF, Mashreky SR, Eriksson U-B. Bangladeshi school-age children’s experiences and perceptions on child maltreatment: A qualitative interview study. Child Care Health Dev. 2017;43(6):876–83. 10.1111/cch.12508 28871592

[12] Malik F. Determinants of child abuse in Pakistani families: parental acceptance-rejection. Int J Bus Soc Sci. 2010;1(1):67–80.

[13] Unicef. Violence against children in East Asia and the Pacific: A regional review and synthesis of findings. Bangkok; 2014. (Strengthening Child Protection Systems Series). Report No.: 4. Available from: http://www.unicef.org/eapro/Violence_against_Children_East_Asia_and_Pacific.pdf Cited 27 Oct 2017

[14] UBS Optimus Foundation. Promoting research to prevent child maltreatment. Geneva, Switzerland.; 2012. Available from: http://www.who.int/violence_injury_prevention/violence/child/ispscan_report_june2013.pdf Cited 13 Jan 2017

[15] Kumar MT, Kumar S, Singh SP, Kar N. Prevalence of child abuse in school environment in Kerala, India: An ICAST-CI based survey. Child Abus Negl. 2017;70:356–63. 10.1016/j.chiabu.2017.06.025 28692832

[16] de Zoysa P. A study on parental disciplinary practices and an awareness program to reduce corporal punishment and other forms of negative parental practices. 2013. Available from: http://www.cpcnetwork.org/wp-content/uploads/2014/04/Report-on-Corporal-Punishment.pdf Cited 25 March 2018

[17] Bureau of Statistics Punjab, Planning & Development Department; Government of the Punjab, UNICEF Punjab. Multiple Indicator Cluster Survey, Punjab 2014, Final report, Lahore, Pakistan: Bureau of Statistics Punjab, Planning & Development Department; Government of the Punjab, UNICEF Punjab; 2016.

[18] Trang NHM, Duc NHC. Violent disciplinary practices towards children among caregivers in Vietnam: A cross-sectional survey. Eur J Soc Sci. 2014;43(4):305–13.

[19] Hadi A. Child abuse among working children in rural Bangladesh: prevalence and determinants. Public Health 2000;114(5):380–4. 10.1038/sj.ph.1900664 11035460

[20] Biswas A., Rahman A., Mashreky S., Rahman F., Dalal K. Unintentional injuries and parental violence against children during flood: A study in rural Bangladesh. Rural Remote Health. 2010;10(1199):37. Epub 2010 Mar 16. 20337500

[21] Kabir M. The state of violence against children in Bangladesh. Dhaka; Center for studies on human rights law, September 2001. (No. 123). Dhaka.

[22] Unicef. Baseline study: Children in Bangladesh News Media. Dhaka, Bangladesh; 2010. Available from: http://www.unicef.org/bangladesh/knowledgecentre_7018.htm Cited 16 January 2017

[23] Haque MA, Janson S, Moniruzzaman S, Rahman AF, Islam SS, Mashreky SR, et al. Child maltreatment portrayed in Bangladeshi newspapers. 2018 (Author's manuscript submitted for publication)

[24] Bangladesh Bureau of Statistics and Unicef Bangladesh. Child well-being survey in urban areas of Bangladesh, key results. Dhaka, Bangladesh; 2016. Available from: https://www.unicef.org/bangladesh/CWS_in_urban_areas_Key_Findings_Report_Final_04122016.pdf Cited 13 January 2017

[25] Belsky J. Child maltreatment: An ecological integration. Am Psychol. 1980;35(4):320–35. 10.1037//0003-066x.35.4.320 7386966

[26] Doidge JC, Higgins DJ, Delfabbro P, Segal L. Risk factors for child maltreatment in an Australian population-based birth cohort. Child Abuse Negl. 2017;64:47–60. 10.1016/j.chiabu.2016.12.002 28027464

[27] Krug EG, Dahlberg LL, Mercy JA, Zwi AB, Lozano R. The world report on violence and health. Geneva, World Health Organization; 2002. Available from: http://www.who.int/violence_injury_prevention/violence/world_report/en/full_en.pdf?ua Cited 14 April 2018

[28] Lwanga SK, Lemeshow S. Sample size determination in health studies A practical manual. World Health Organization 1991.

[29] Runyan D, Brandspigel S, Zolotor A, Dunne M. Manual for Administration: The ISPCAN Child Abuse Screening Tool (ICAST). West Chicago: ISPCAN; 2015.

[30] Hein IM, De Vries MC, Troost PW, Meynen G, Van Goudoever JB, Lindauer RJL. Informed consent instead of assent is appropriate in children from the age of twelve: Policy implications of new findings on children’s competence to consent to clinical research. BMC Med Ethics. 2015;16(1):76 Available from: 10.1186/s12910-015-0067-z 26553304 PMC4640170

[31] Al-Eissa MA, Saleheen HN, Al Madani S, Al Buhairan FS, Weber A, Fluke JD, et al Determining prevalence of maltreatment among children in the kingdom of Saudi Arabia. Child Care Health Dev. 2016;42(4):565–71. 10.1111/cch.12325 Epub 2016 Feb 15. 26879326

[32] Ribeiro IMP, Teixeira Ribeirol ÁS, Pratesil R, Gandolfil L. Prevalência das várias formas de violência entre escolares (Prevalence of various forms of violence among school students). ACTA Paul Enferm. 2015;28(1):54–9. 10.1590/1982-0194201500010

[33] Chang HY, Lin CL, Chang YT, Tsai MC, Feng JY. Psychometric testing of the Chinese version of ISPCAN Child Abuse Screening Tools Children’s Home Version (ICAST-CH-C). Child Youth Serv Rev. 2013;35(12):2135–9. 10.1016/j.childyouth.2013.10.020

[34] Harris PA, Taylor R, Thielke R, Payne J, Gonzalez N, Conde JG. Research electronic data capture (REDCap)-A metadata-driven methodology and workflow process for providing translational research informatics support. J Biomed Inform. 2009;42(2):377–81. 10.1016/j.jbi.2008.08.010 18929686 PMC2700030

[35] Filmer D, Pritchett L. Estimating wealth effects without expenditure data—or tears: an application to educational enrollments in States of India. Demography. 2001;38(1):115–32. 10.1353/dim.2001.0003 11227840

[36] Al-Eissa MA, Al Buhairan FS, Qayad M, Saleheen H, Runyan D, Almuneef M. Determining child maltreatment incidence in Saudi Arabia using the ICAST-CH: A pilot study. Child Abus Negl. 2015;42:174–82. 10.1016/j.chiabu.2014.08.016 25220480

[37] Sofuoglu Z, Oral R, Aydin F, Cankardes S, Kandemirci B, Koc F, et al Epidemiological study of negative childhood experiences in three provinces of Turkey. Türk Pediatr Arşivi. 2014;49(1):47–56. 10.5152/tpa.2014.838 26078632 PMC4462266

[38] Nikolaidis G, Petroulaki K, Zarokosta F, Tsirigoti A, Hazizaj A, Cenko E, et al Lifetime and past-year prevalence of children’s exposure to violence in 9 Balkan countries: THE BECAN study. Child Adolesc Psychiatry Ment Health. 2018;12(1). 10.1186/s13034-017-0208-x 29308086 PMC5749026

[39] Stoltenborgh M, Bakermans-Kranenburg MJM, van IJzendoorn MHM, Alink LLRA. Cultural-geographical differences in the occurrence of child physical abuse? A meta-analysis of global prevalence. Int J Psychol. 2013;48(2):81–94. 10.1080/00207594.2012.697165 23597008

[40] Fang X, Fry DA, Ji K, Finkelhor D, Chen J, Lannen P, et al The burden of child maltreatment in China: A systematic review. Bull World Health Organ. 2015;93(3): 176–185C. 10.2471/BLT.14.140970 25838613 PMC4371492

[41] Ji K, Finkelhor D. A meta-analysis of child physical abuse prevalence in China. Child Abus Negl. 2015;43:61–72. 10.1016/j.chiabu.2014.11.011 25498804

[42] Beazley H, Bessell S, Ennew J, Waterson R. What children say: Results of comparative research on the physical and emotional punishment of children in Southeast Asia and the Pacific, 2005 Stockholm: Save the Children; 2006.

[43] Mookherjee N. 'Remembering to forget’: public secrecy and memory of sexual violence in the Bangladesh war of 1971. J R Anthr Inst. 2006;12(2):433–50. 10.1111/j.1467-9655.2006.00299.x

[44] Cicchetti D, Toth SL, Maughan A. An Ecological-Transactional Model of Child Maltreatment In: Sameroff AJ, Lewis M, Miller SM, editors. Handbook of Developmental Psychopathology. Boston, MA: Springer US; 2000 p. 697.

[45] Daral S, Khokhar A, Pradhan S. Prevalence and determinants of child maltreatment among school-going adolescent girls in a semi-urban area of Delhi, India. J Trop Pediatr. 2016;62(3):227–40. 10.1093/tropej/fmv106 26769624

[46] Irfan S, Cowburn M. Disciplining, chastisement and physical child abuse: perceptions and attitudes of the British Pakistani community. J Muslim Minor Aff. 2004;24(1):89–98. 10.1080/1360200042000212151

[47] Chen JK, Wei HS. Student victimization by teachers in Taiwan: prevalence and associations. Child Abus Negl. 2011;35(5):382–90. 10.1016/j.chiabu.2011.01.009 21620165

[48] Theoklitou D, Kabitsis N, Kabitsi A. Physical and emotional abuse of primary school children by teachers. Child Abuse Negl. 2012;36(1):64–70. 10.1016/j.chiabu.2011.05.007 22197151

[49] Deb S, Modak S. Prevalence of violence against children in families in Tripura and its relationship with socio-economic factors. J Inj Violence Res. 2010;2(1):5–18. 10.5249/jivr.v2i1.31 21483193 PMC3134897

[50] Lakhdir MPA, Farooq S, Khan UR, Parpio Y, Azam SI, Razzak J, et al Factors associated with child maltreatment among children aged 11 to 17 years in community settings of Karachi, Pakistan, using Belsky Ecological Framework. J Interpers Violence. 2017;886260517726973 10.1177/0886260517726973 29294892

[51] Unicef. Opinions of children of Bangladesh on corporal punishment: children’s opinion poll 2008. 2008. Available from: https://www.unicef.org/bangladesh/Opinion_Poll_2009.pdf Cited 16 January 2017

[52] Flynn-O’Brien KT, Rivara FP, Weiss N, Lea V, Marcelin L, Vertefeuille J, et al Prevalence of physical violence against children in Haiti: A national population-based cross-sectional survey. Child Abus Negl. 2015;51:154–62. 10.1016/j.chiabu.2015.10.021 26612595 PMC5928512

[53] Huda T. Why child domestic workers are prime victims. The Daily Star. 2017 Jul 10; Available from: https://www.thedailystar.net/opinion/human-rights/why-child-domestic-workers-are-prime-victims-1430632 Cited 24 Oct 2018

[54] Benbenishty R, Zeira A, Astor RA. Children’s reports of emotional, physical and sexual maltreatment by educational staff in Israel. Child Abus Negl. 2002;26(8):763–82. 10.1016/S0145-2134(02)00350-2 12363330

[55] Cappa C, Dam H. Prevalence of and risk factors for violent disciplinary practices at home in Viet Nam. J Interpers Violence. 2014;29(3):497–516. 10.1177/0886260513505215 24162756

[56] Hunter WM, Jain D, Sadowski LS, Sanhueza AI. Risk factors for severe child discipline practices in rural India. J Pediatr Psychol. 2000;25(6). 10.1093/jpepsy/25.6.435 10980048

[57] Zolotor AJ, Theodore AD, Coyne-Beasley T, Runyan DK. Intimate partner violence and child maltreatment: Overlapping risk. Br Treat Cris Interv. 2007;7(4):305–21. 10.1093/brief-treatment/mhm021

[58] Rumm PD, Cummings P, Krauss MR, Bell MA, Rivara FP. Identified spouse abuse as a risk factor for child abuse. Child Abus Negl. 2000;24(11):1375–81. 10.1016/S0145-2134(00)00192-7 11128171

[59] Jernbro C, Janson S. Violence against children in Sweden 2016 The Children’s Welfare Foundation, Sweden Stockholm; 2017.

[60] Smith Slep AMS, O’Leary SG. Examining partner and child abuse: Are we ready for a more integrated approach to family violence? Clin Child Fam Psychol Rev. 2001;4(2):87–107. 10.1023/A:1011319213874 11771795

[61] Stith SM, Liu T, Davies LC, Boykin EL, Alder MC, Harris JM, et al Risk factors in child maltreatment: A meta-analytic review of the literature. Aggress Violent Behav. 2009;14(1):13–29. 10.1016/j.avb.2006.03.006

[62] Bangladesh Bureau of Statistics Statistics division, Ministry of Planning. Report of the household income & expenditure survey 2010. Bangladesh Bureau of Statistics, Dhaka. 2011. Available from: http://203.112.218.65:8008/WebTestApplication/userfiles/Image/LatestReports/HIES-10.pdf Cited 7 Feb 2018

[63] Gorjian Z, Mahmoodi N, Nabhani N, Mohseni A, Baghbani M, Zahedi A, et al The relationship between child abuse and family, psychological and social factors among children. J Fundam Appl Sci. 2017;9(3):1735–47. 10.4314/jfas.v9i3.31

